# A new CO_2_ laser technique for the treatment of pediatric hypertrophic burn scars: An observational study

**DOI:** 10.1097/MD.0000000000005168

**Published:** 2016-10-21

**Authors:** Tomasz Żądkowski, Paweł Nachulewicz, Maciej Mazgaj, Magdalena Woźniak, Czesław Cielecki, Andrzej Paweł Wieczorek, Iwona Beń-Skowronek

**Affiliations:** a Department of Paediatric Surgery; b Department of Paediatric Radiology; c Department of Endocrynology, Medical University of Lublin, Lublin, Poland.

**Keywords:** burn, children, CO_2_, laser, scar

## Abstract

Treatment of hypertrophic scars arising as a result of thermal burns in children is still a big problem. The results of the treatment are not satisfactory for patients and parents, and new methods of treatment are still investigated.

We present the use of one of the most modern carbon dioxide (CO_2_) lasers (Lumenis Encore laser equipped with a Synergistic Coagulation and Ablation for Advanced Resurfacing module) in the treatment of hypertrophic scars in children after burns.

From March to April of 2013, a group of 47 patients aged 6 to 16 years underwent 57 laser surgery treatments. The average time from accident was 7.5 years. The results of treatment were investigated in 114 areas. The assessed areas were divided into 2 groups: 9-cm^2^ area 1, where the thickness of the scar measured by physician was the lowest and 9-cm^2^ area 2, where the thickness of the scar was the biggest. The results were considered on the Vancouver Scar Scale (VSS) independently by the surgeon and by parents 1, 4, and 8 months after the procedure. In addition, ultrasound evaluation of the scar thickness before and after laser procedure was made.

VSS total score improved in all areas assessed by both the physician and parents. The biggest change in total VSS score in area 1 in the evaluation of the investigator was obtained at follow-up after the 1st month of treatment (average 7.23 points before and 5.18 points after the 1st month after surgery—a difference of 2.05 points). Scar ratings by parents and the physician did not differ statistically (*P* < 0.05). In the ultrasound assessment, the improvement was statistically significant, more frequently for both minimum and maximum thickness of the scars (B-mode measures) (*P* < 0.05).

The use of a CO_2_ laser in the treatment of hypertrophic scars in children is an effective and safe method. The use of a CO_2_ laser improves the appearance and morphology of scarring assessed using the VSS by both the parents and the physician. The treatment also reduced the thickness of scars evaluated by ultrasound.

## 1 Introduction

Treatment of hypertrophic scars arising as a result of thermal burns in children is still an unresolved problem, and to improve its performance is still a challenge due to very complex morphology of these scars. Results of treatment are still not satisfactory for patients and parents.^[1,2]^ In the process of burn wound healing, hypertrophic scars arise in many cases and are found in over 20% of burned children under 5 years of age.^[3–5]^ The appearance of the scar may be described using one of the standardized rating scales. The most commonly used is the Vancouver Scar Scale (VSS), which evaluates 4 parameters: pigmentation, height, pliability, and vascularity.^[6]^ Another sensitive and reproducible method of estimation is ultrasonography (US). US allows for evaluation of the thickness of the scar tissue but also the morphology of the tissue lying below the scar.^[7,8]^

Contemporary techniques of treatment are injections of corticosteroids, topical application of silicone (silicone patches and silica gel), debridement, ointment with vitamin A, radiotherapy, and, recently, lasers.^[9–13]^ In the treatment of hypertrophic scars, high-energy lasers are used. A pulsed-dye laser (PDL) is used for the treatment of immature scars when the process of scar maturation has not yet finished.^[14–19]^ In contrast, a carbon dioxide (CO_2_) laser is effective in the treatment of mature scars when at least 1 year has passed from the trauma burn injury.^[20–23]^ In 2012, the company Lumenis introduced a modification to the high-energy CO_2_ laser (UltraPulse® Encore; Lumenis, Santa Clara, CA, USA) by adding a module for the treatment of scars—Synergistic Coagulation and Ablation for Advanced Resurfacing (SCAAR FX). The device generates hundreds of very deep microchannels that intersect haphazardly to arrange collagen fibers of highly contracted scars. Even after the 1st procedure, patients feel a reduction of tension and softening of the scar. The minimal coagulation zone around the ablative channels reduces inflammatory processes and overreaction of the tissue to the laser pulse.^[24]^

The use of lasers in the treatment of various skin conditions has been widely reported. Light amplification by stimulated emission of radiation (Laser) is a type of electromagnetic energy, which can be precisely focused on a specific lesion. Lasers may be differentiated from regular light by 3 characteristics: coherence, monochromaticity, and collimation. The active medium is stimulated by an external power source that results in the generation of photons in a reflecting chamber. When the photon or light energy comes into contact with a particle, it may be absorbed, reflected, or transmitted. A particle is stimulated only when the light is absorbed.^[25–28]^

High-power lasers are defined as lasers with a power output of 500 mW or more (applied for surgical purposes).^[12,20,21,25]^ Low-power lasers range from 1 mW to up to 500 mW and are generally used for therapeutic purposes such as tissue repair and pain management. In this category, the following types are included:

- gaseous medium: argon, helium–neon, CO_2_, and krypton;
- semiconductor medium: gallium arsenide, gallium aluminium, and gallium aluminium arsenide; and
- crystalline medium: neodymium–yttrium aluminium garnet and ruby.^[14]^

It is assumed that lasers coagulate capillaries causing local hypoxia, releasing lactic acids, and decreasing pH and a2-macroglobulin concentration, thereby enhancing lysis of collagen.^[12,25]^ Satisfactory results were reported in about 75% of abnormal scars that were treated.^[12,20–23]^

The choice of scar treatment is undertaken individually for each patient depending on the time elapsed since its inception, location, presence of additional deformation, thickness, type of scar, and contraindications to the use of a specific action. For these reasons, there are no standardized methods of treatment that would be universal for all types of scars, but laser therapy is finding its place as one of the accepted methods, although its efficacy needs further long-term evaluation.

## 2 Material and methods

Before starting the investigation, approval was obtained from the Medical University of Lublin Ethics Commission (KE-0254/299/2012). From 2004 to 2007, 550 burned children were treated in the Department of Paediatric Surgery and Traumatology at the Medical University of Lublin. Of those invited to participate in the investigation, 120 patients responded. Inclusion criteria passed 47 patients, 21 boys and 26 girls aged 7 to 16 years (mean age 10.5 years). The average time from burns was 7.5 ± 2 years (age of the patients at the day of therapy). Average burned total body surface area was 8.8%, with a standard deviation (SD) of 8%. Minimum and maximum values were 1% and 42%, respectively.

The inclusion criteria aimed to minimize the need for unnecessary analgesic procedures such as general anesthesia or multimodal therapy. The exclusion criteria minimized the risk of adverse events during and after the treatment that could be connected with poor social and living conditions or any coexisting chronic diseases. The criteria are presented in Table 1.

**Table 1:**
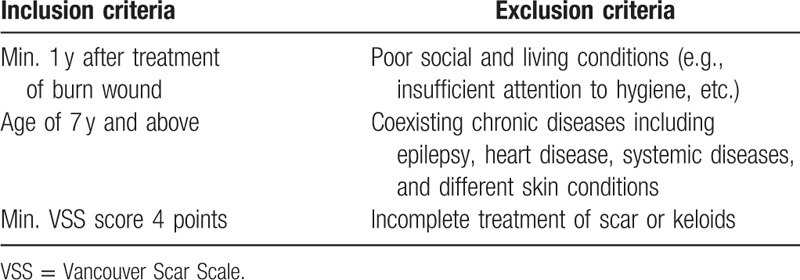
Inclusion and exclusion criteria of the study.

**Figure 1:**
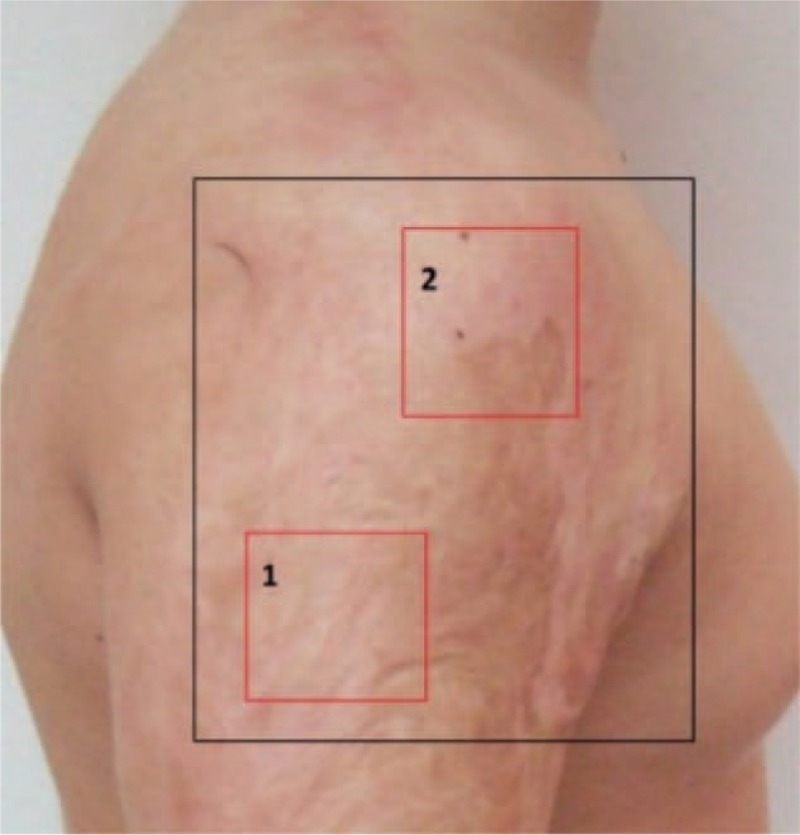
Black square shows the treatment area with areas 1 and 2 taken for the evaluation (red squares).

In the analyzed group of 47 patients, 57 laser sessions were performed (in 10 patients, due to extensive burn scars, the procedure was performed twice). Before laser therapy, there were 114 localized areas which were assessed by the physician, parents, and US. The investigated areas were divided into 2 groups. The 1st area included the border of the scar and intact skin and was designated as area 1. The 2nd area included the most advanced visual portion of the change and was designated as area 2. The surface area was a single square of 3 cm × 3 cm (9 cm^2^—the surface of the area was equal to the surface of the laser head) (Fig. 1).

The questionnaire according to VSS assessment was fulfilled by the physician and parents (Table 2). Any previous clinical finding for the evaluation of VSS was hidden from both physician and parents.

**Table 2:**
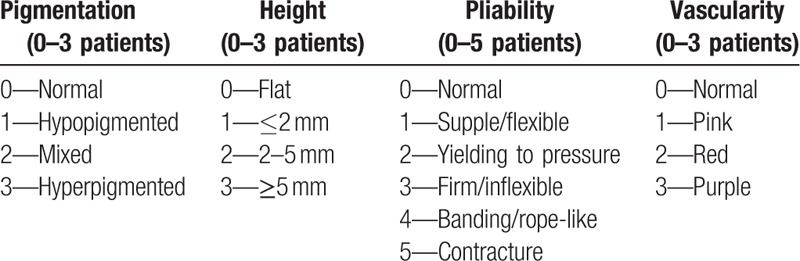
VSS.

Primary evaluation in the analyzed group revealed the highest scores in pigmentation and pliability parameters. We found over 90% of patients with mixed or hyperpigmentation (scores 2 and 3) and over 90% of patients with the highest pliability of scars (scores >2) (Table 3).

**Table 3:**
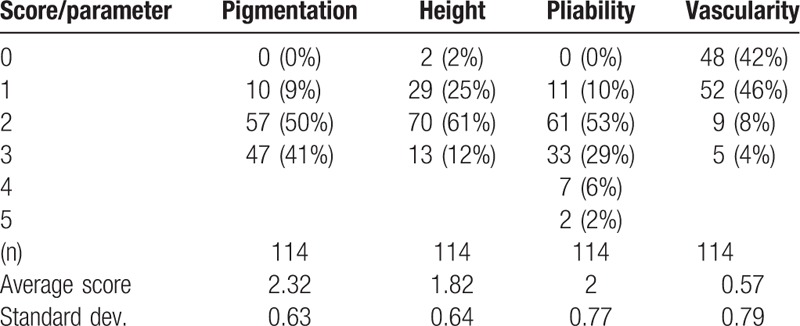
VSS evaluation before treatment by physician.

In follow-up, the physician assessed all 114 areas; parents assessed only the 57 in area 2. The assessments were performed before and 1, 4, and 8 months after laser therapy. If the parameter before surgery was estimated at 3 points, and 8 months after treatment the same parameter was estimated at 2 points, the result had improved. If the given parameter before treatment was assessed as 2 points, and after 8 months earned 3 points, the result was assessed as deterioration. For patients whose score in the last inspection after 8 months was the same as before the surgery, the results did not change. Also, before therapy and during control visits, US assessment of scar thickness was performed.

Before surgery, 47 patients had undergone US assessment of scars in 114 areas eligible for surgery. US was performed in both areas. A Philips iU22 (Philips Healthcare, Andover, MA, USA) US device equipped with an electronic broadband L12-5 linear head was used. Default settings were set for “Small Parts Superficial”. Focusing was established in the near field image at a depth corresponding to the boundary of the skin and subcutaneous tissue (i.e., 3–4 mm). Priority was set to the image resolution and gain of 60%.

**Figure 2:**
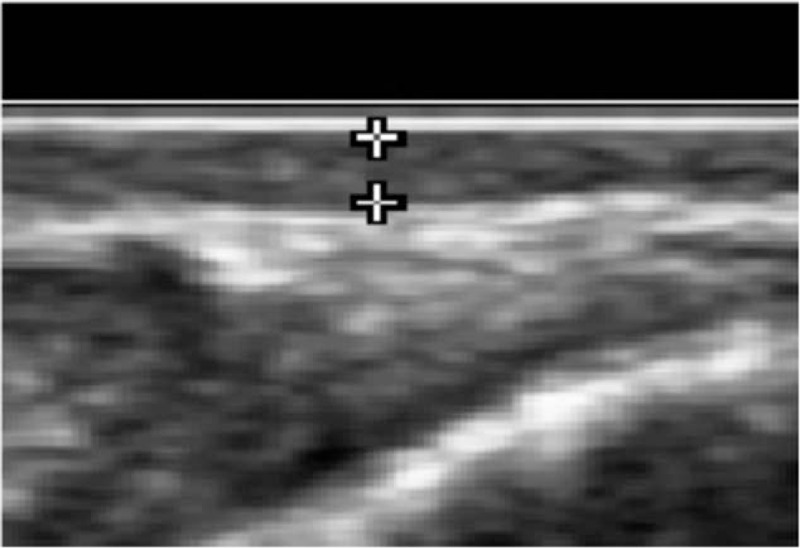
B-mode ultrasound measuring maximum scar thickness. Scar and subcutaneous tissue are visible in the image as a superficially limited hypoechoic layer and from the deep hyperechoic bands.

US calculation measured the distance between the inner edges of the hyperechoic limitations. The maximum thickness of the scar was defined as the result of measurement of the thickest places in the selected scar areas. The smallest thickness of the scar was one of 2 possible values: either the thinnest place surrounded by fragments of thicker scars in the case of irregular scars or thick scarring at the point of transition to the surrounding healthy skin in scars with regular contours and a spindle section (B-mode maximum and minimum thickness) (Fig. 2).

### 2.1 Laser procedure

5% prilocaine/lidocaine cream (EMLA®) cream (Astra Zeneca, Mississauga, ON, Canada) was applied topically 75 minutes before laser procedure; 60 minutes before procedure, patients were administered paracetamol intravenously (15 mg/kg); and then 30 minutes before procedure, morphine (0.15 mg/kg). In the operating theater, the EMLA dressing was removed and the treated area was prepared with Octenisept solution (Octenidine Dihydrochloride [Octenisept, Schulke and Mayr GmBH, Norderstedt, Germany] and phenoxyethanol solution, Schulke) as for surgery. Before performing the treatment, patients were connected to a face mask with an oxygen flow of 2 to 3 L/min, and a bolus of midazolam was administered intravenously in a dose of 0.1 mg/kg body weight.

**Figure 3:**
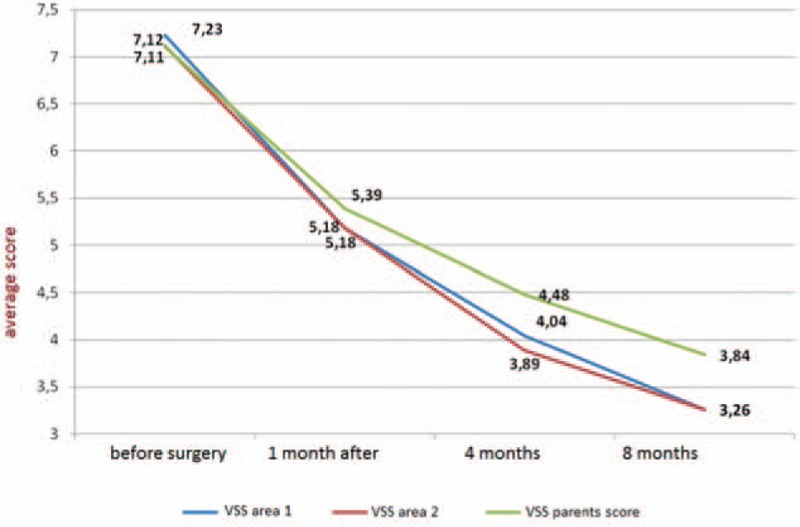
Average results for Vancouver Scar Scale scores before and after treatment. Areas 1 and 2 evaluated by the physician, area 2 only by parents.

The procedure was performed using a Lumenis UltraPulse Encore CO_2_ laser equipped with a SCAAR FX module and 2 heads—Deep FX (SCAAR FX mode) and CPG (Active FX mode). The treatment consisted of 3 phases (Table 3). In the initial phase, abrasion of wound edges was done in 19 procedures (33%) with the laser head in CPG Active FX mode. During this procedure, total energy of 30 to 40 mJ with a pulse frequency of 300 to 450 Hz was used. During the next stage, the Deep FX SCAAR mode was used in all 57 procedures. The laser beam energy was used perpendicularly to the surface of the skin and under an angle of 45° (Fig. 3). The mean energy value used was 119 mJ (range from 60 to 150 mJ, SD 29 mJ). Patients with less-advanced scars were eligible for treatment with lower doses of energy than those with a high progression of changes. In the last stage of the procedure, a mild abrasive mode—FX ACTIVE—was used. A mild abrasion of the treated surface with energy of 60 to 100 mJ and frequency of 75 to 125 Hz was used. This part of the procedure was performed in 57% (n = 26) of patients.

After the procedure, an occlusive dressing was applied. The treated surface was covered with ointment with allantoin, and additionally protected with gauze soaked in liquid Octenisept. Ice packs were applied topically, and intravenous analgesics were administered (paracetamol and morphine) in patients who reported pain. On the 2nd postoperative day, the dressing was changed, and the patient was discharged home. Ambulatory control visits took place on the 7th and 14th days after surgery until the healing process was finished.

In the statistical evaluation of the results, the Statistica 7.0 (StatSoft Inc., Tulsa, USA) program was used. In the case of qualitative data, a nominal scale was used and its particular kind, if the answer was yes/no, was a dichotomous scale. Quantitative data were presented on an ordinal scale. When analyzing age, US measurements, and VSS measurements, scale ranges were used. To compare quantitative data, the range of values (min. and max.), arithmetic mean, SD, median, and frequency tables were used.

All the tested variables were analyzed for normal distribution using the Shapiro–Wilk test. The homogeneity of variance in case of confirmation of normality was confirmed by Levene test. For certain assumptions of normality and homogeneity of variance, ANOVA and *U* test were used. In cases where at least one of the assumptions was violated, the study used nonparametric test counterparts, namely ANOVA, Kruskal–Wallis (ANOVA), and Mann–Whitney *U* test (*U* test). To compare the incidence in different groups, tables bisected the significance of the parameters evaluated based on a chi-squared test. In the paper, the confidence interval was set at 5%, and therefore the significance of the phenomena occurred at *P* < 0.05.

## 3 Results

In all 114 treated areas, wound healing was completed up to 14 days after surgery. Observed long-lasting complications after surgery were erythema, discoloration, sensitization, and overgrowth (Table 3). One month after procedure, complications occurred in 37% of all the considered areas (n = 114) but in the 8th month after surgery was observed only in 9% of patients. The most common complication after surgery was erythema, which accounted for 67% of all complications. Erythema after treatment occurred in 28 areas, and after 8 months of follow-up was still visible in 2 areas. Blemishes and scar excesses occurred in 3 areas studied, and during follow-up in only 1 case had the condition improved (Table 4).

**Table 4:**
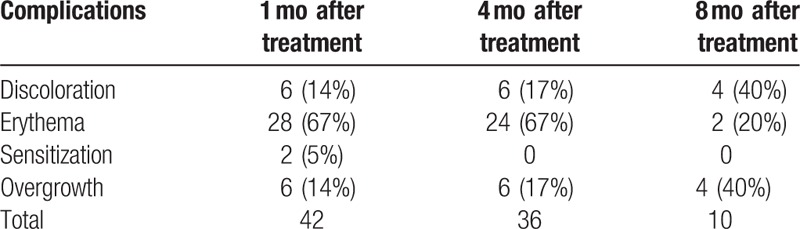
Distribution of post-treatment complications.

All parameters of VSS were analyzed as overall results and separately for all 4 parameters. The overall results assessed by parents and physician did not differ statistically (*P* < 0.05) and were comparable. The total score obtained on the VSS as a whole was improved in all areas, both in the judgment of the physician and parents. The biggest changes in the assessment of scarring were observed 1 month after surgery, but after 8 months of observation later improvements were observed (Fig. 3).

*Pigmentation*—in all patients before treatment, abnormal pigmentation of the skin was observed, both hypo- and hyperpigmentation or miscellaneous changes. Improvement in pigmentation of the scars was observed in 81% of assessed area 1 and no worsening in valuation was observed by a physician (*P* < 0.05). In assessment by parents, the results were similar and did not differ statistically (*P* < 0.05) (Fig. 3).

*Height of the scar*—improvement in the height parameter after procedure was observed in 88% of assessed areas (*P* < 0.05).

*Pliability*—the majority of patients (98%) had improved scar pliability after surgery (*P* < 0.05).

**Figure 4:**
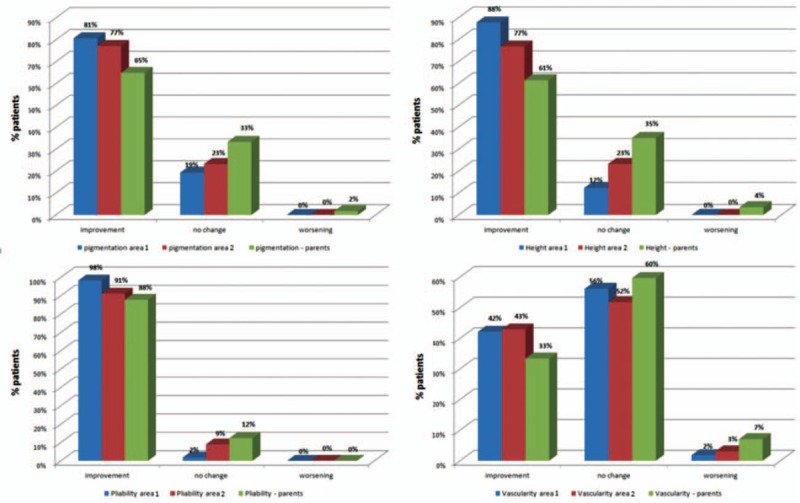
Vancouver Scar Scale parameter score improvement in areas 1 and 2 by physician and area II by parents—all 4 parameters (pigmentation, height, pliability, and vascularity).

*Vascularity* was the only scar parameter for which no significant statistical improvement was observed (*P* > 0.05). In the evaluation by a physician, a large number of cases did not cause changes in vascularity (56% in area 1), and improvement was observed only in 42% of area 1. It was also the only evaluated parameter in which deterioration was observed in area 1. All specific data are presented in Fig. 4.

**Figure 5:**
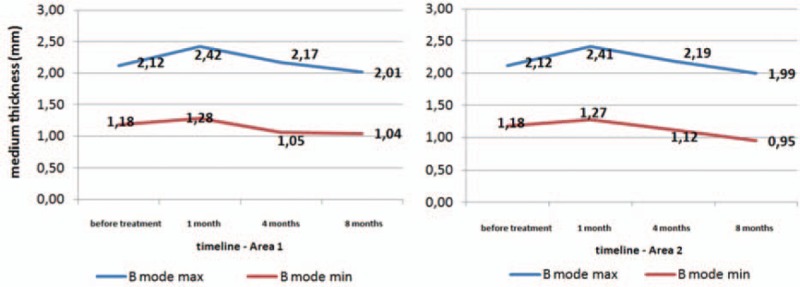
Average results of ultrasonography measurements (thickness) before and after treatment.

US investigation found that in area 1 improvement was almost twice as likely as deterioration (56% improvement vs 28% worsening) (*P* = 0.0516). For area 2, improvement in the US thickness range—for B-mode max. and min.—was almost 3 times more prevalent than deterioration (62% improvement vs 24% worsening). Improvement was statistically more frequent for both minimum and maximum thickness of scars (B-mode measures) (*P* < 0.05). The average US thickness measurement findings before treatment and during follow-up for both areas are shown in Fig. 5.

**Figure 6:**
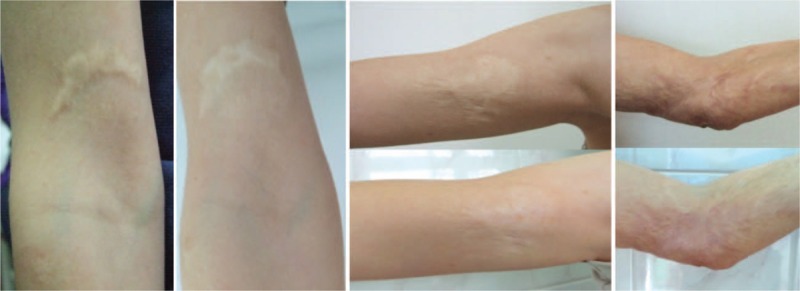
Examples of results before treatment and after 8 months: left picture—right arm burn scar, center picture—right arm burn scar, and right picture—left elbow burn scar.

Exemplary results of treatment before and after laser procedure are shown in Fig. 6. No additional interventions during procedure or follow-up period were performed.

## 4 Discussion

Traditional treatment of burn scars with injections of corticosteroids, vitamin A and onion extract ointments, radiotherapy, pressure therapy, and drug therapy using bleomycin and verapamil have nowadays mostly only historical value.^[26–41]^ Currently, widely used silicone patches and dressings have the greatest effectiveness in preventing scar overgrowth but also help reduction of hypertrophy in formed scars.^[42–48]^ The rapid development of laser technology and the increasingly wider range of applications of it in various medical indications results in it replacing other, often ineffective, methods of treatment. The most spectacular example is the use of PDL and CO_2_ lasers in the treatment of hypertrophic scars, where effectiveness has been proven in many clinical studies.^[49–58]^ The largest group of patients was described by Kawęcki et al. The study included 327 patients aged 3 to 80 years who underwent 592 procedures using a Derma K laser (combination of Er:YAG and CO_2_); 223 patients were those with burn scars.^[12]^ Donelan et al treated 57 patients aged 2 to 21 years (mean age 13 years) using PDL (V-beam, Candela, Candela Corp.; Wayland, CA, USA). In that group, 61% of patients had flame burns and 25% had contact burns. The time from burn accident was 2 to 199 months (average 64 months).^[50]^ Bernstein et al^[59]^ performed treatments using a CO_2_ laser (UltraPulse, Coherent and SilkTouch, Sharplan Laser Corp.) on 30 patients aged 14 to 84 years (mean age 54.3 years). Other authors also presented groups of patients heterogeneous in terms of age and scar type.^[59–65]^

The group of patients in our study was homogeneous. The youngest patient was 7 years old, the oldest 16 (median 10 years). The average time from burns was 7.5 ± 2 years, but in 84% (n = 48), the time from burns was between 6 and 9 years. The patients were treated with a CO_2_ Lumenis UltraPulse laser equipped with 2 heads, Deep FX and CPG, and a SCAAR FX module and underwent a standardized surgical procedure.

Qu et al also used a Lumenis UltraPulse CO_2_ laser in the Active FX (CPG head) operating mode with a mild abrasive surface energy of 80 to 100 mJ, density 2% to 4%, at frequencies of 200 Hz. Deep exposure (Deep FX head) was done with an average energy of 20 mJ, 300 Hz, with a density of 10% to 15%. During this investigation, the SCAAR FX module was not yet available. The procedures were repeated 3 to 4 times in each patient.^[60]^ In our study, we performed only a single treatment on all treated surfaces, additionally using the SCAAR FX module.

One of the most difficult problems in the assessment of the real effects of treatment is comparison of the results. Bae and Bae^[66]^ in their meta-analysis showed that the Patient and Observer Scar Assessment Scale (37% of all reports) and VSS (34%) are most often used in valuation of results. In reports assessing the effectiveness of laser scar correction therapy, the VSS (basic and modified) is used most often.^[14,59,60,63,66,67]^ In our method, we used primary VSS and US. We were not able to find research on the outcomes of treatment of scars evaluated by US in the present literature.

Past results showed that treatment of mature hypertrophic scars after burns using a fractional CO_2_ laser is effective.^[12,20,24,55,59,62,63,67]^ The effect of treatment is to change 1 or more of the parameters characterized by scarring, such as abnormal pigmentation, scars, eminence above the level of intact skin, cohesion, and vascularization. These parameters assessed in VSS determine the outcome of the surgery. During evaluation of our results, we compared not only total results in VSS but also the results of individual elements of the scale, where we could obtain information about improvement, lack of change, and deterioration of the evaluated elements. Judging the results, the largest differences were observed in pigmentation before surgery and after 8 months (7.23 vs 3.26, a difference of 3.97 points). Comparable results were also obtained when assessment was performed by parents; however, results showed a smaller difference (7.11 vs 3.84—a difference of 3.27 points) but comparison of the results between doctor and parents did not differ statistically (*P* < 0.05).

Hultman et al^[65]^ in their study showed average improvement on the VSS of 5.27 points (10.43 points before vs 5.16 points after surgery). The study used different methods of treatment using a PDL in patients with highly vascularized scars, UltraPulse CO_2_ laser (using Active FX and Deep FX modes) in patients with hypertrophic scars, and a diode laser (IPL for staining and Alexandrite laser) in patients with keratosis pilaris. The authors emphasize the potentially biased nature of the measurements according to the VSS despite hiding previous research results in a subsequent control.^[58,63,67]^ In our study, we also hid the results of previous measurements, both from parents and the physician. We also reasoned, like Hultman et al, that the assessment made by VSS after surgery could be hidden by a physician due to expectations of positive outcomes of the research project, but because of the experimental nature of the study we did not choose to apply a double masking.^[65]^

This study included an analysis of the effects of the use of a single-laser procedure. Kawęcki et al performed 2 to 5 treatment sessions in each case.^[12]^ The choice of a single treatment procedure was connected with the several times higher energy used in our study in the SCAAR FX mode; we usually used a maximal pulse energy value of 150 mJ.

Hultman et al said that the greatest improvement in VSS was observed after the 1st procedure, evaluated after 1 month (10.43 vs 6.67 points). Subsequent treatment sessions repeated every 4 to 6 weeks showed minor improvements. Checks 1 month after the 1st treatment noted a difference in mean score of 3.76.^[35]^ In our results, changes in VSS between 4 and 8 months were the smallest and were connected with scar stabilization after laser therapy. Ozog et al, in their study using an UltraPulse CO_2_ laser, obtained 100% (n = 10) improvement, and the change of mean VSS score was 3.6 points.^[61]^ Others reported similar effects of treatments assessed by dedicated standardized scales.^[59–63,67]^ Assessing both our results and those of other researchers, we may conclude that that CO_2_ lasers are very effective in the treatment of hypertrophic scars and in many studies reached almost 100% improvement.

One of the parameters measured in VSS is thickness of the scar, but we may assess only the portion located above the level of the surrounding skin, and this measurement is subjective. Scar thickness was measured by US in our study. To our knowledge, no data on evaluation of scar thickness by US after laser therapy have been published. US is widely used in the diagnosis of skin diseases. The most common applications are evaluation of pathologies such as skin cancer, inflammatory autoimmune processes (scleroderma, lupus, and hidradenitis suppurativa), and benign skin cancers.^[8,64]^ Some authors use US to assess the degree of burns and burn scar monitoring.^[7,8]^ Wang et al in their study measured the thickness of scars using B-mode US 3, 6, and 9 months after burns. They showed the presence of a thick scar peak at 6 months after surgery. Average measurements at 3 months were 0.3 cm (min. 0.2 cm and max. 0.5 cm), at 6 months 0.4 cm (min. 0.2 cm and max. 0.8 cm), and 0.35 cm in the control after 9 months (min. 0.2 cm and max. 0.9 cm).^[7]^ In our study, we performed US before treatment and in the 1, 4, and 8 months after treatment. Comparing results of thickness of scars in all areas (n = 114) before treatment and after 8 months, we found statistical improvement (*P* < 0.05). We noted improvement in 67/114 (59%) measurements in B-mode max. and 74/114 (65%) B-mode min. Scar thickness assessed in the VSS by a physician improved in all cases and in 96% assessed by parents.

The main limitation of this study is that we included only the patients with hypertrophic scars, not all the children with burn scars. We knew that the keloids treatment results would be worse, and if we mixed all the scars, result would not be so great. The methods used and good long-term results have been made possible thanks to the good selection of patients. The other limitation is excluding patients with parents who do not pay enough attention to their children: in order to achieve good long-term outcomes, strict control of the healing process after the procedure is necessary. The healing process takes about 10 to 14 days maximum; therefore, the exclusion criteria rule out the patients with poor social and living conditions: parents who do not care about sufficient assessment, parents who did not respond to previous recommendations, missed checkouts, or do not pay enough attention to hygiene process for their children.

Presently there is no widely accepted standard of treatment of hypertrophic scars and not all methods used are satisfactory for patients and doctors. Application of laser therapy in the treatment of hypertrophic scars is still at a very early stage of development. There is no guidance on which type of laser or energy dose should be used or the frequency of repeated treatments. This is connected with the high volatility of scars, their different location, and morphology. In 1 scar area, we may observe a thick hypertrophic component, keloid formation, or an atrophic area with loss of tissue beneath the scar. CO_2_ laser treatment, in our opinion, is a therapeutic option to be used at least 1 year after burn accidents. In recent years, observed progress in the development of laser technology has resulted in shorter laser beam pulses and generation of higher energy. This change has resulted in a reduction of the burning zone around the site of action and deeper penetration into tissue. It seems that the further development of technologies will allow reduction of the number of complications and increase the effectiveness of treatment. The results obtained in this work are very promising but of course need further evaluation.

## References

[1] De Sousa A Psychological aspects of paediatric burns (a clinical review). *Ann Burns Fire Disasters* 2010; 23:155–159. 21991217 PMC3188258

[2] Bakker A Maertens KJ Van Son MJ Psychological consequences of pediatric burns from a child and family perspective: a review of the empirical literature. *Clin Psychol Rev* 2013; 33:361–371. 23410718 10.1016/j.cpr.2012.12.006

[3] Venter THJ Karpelowsky JS Rode H Cooling of the burn wound: the ideal temperature of the coolant. *Burns* 2007; 33:917–922. 17521815 10.1016/j.burns.2006.10.408

[4] Kobus K Strużyna J Postępowanie zachowawcze I lecznicze dotyczące blizn I zniekształceń pooparzeniowych, zasady rehabilitacji. *Pol Przegl Chir* 2002; 74:655–661.

[5] Jehton J Kulicki M Leczenie oparzeń. *Med Prakt Chir* 2006; 2:27–33.

[6] Brusselaers N Pirayesh A Hoeksema H Burn scar assessment: a systematic review of different scar scales. *J Surg Res* 2010; 164:e115–e123. 20828761 10.1016/j.jss.2010.05.056

[7] Wang XQ Mill J Kravchuk O Ultrasound assessed thickness of burn scars in association with laser Doppler imaging determined depth of burns in paediatric patients. *Burns* 2010; 36:1254–1262. 20573454 10.1016/j.burns.2010.05.018

[8] Kleinerman R Whang TB Bard RL Ultrasound in dermatology: principles and application. *J Am Acad Dermatol* 2012; 67:478–487. 22285673 10.1016/j.jaad.2011.12.016

[9] Berman B Viera MH Amini S Prevention and management of hypertrophic scars and keloids after burns in children. *J Craniofac Surg* 2008; 19:989–1006. 18650721 10.1097/SCS.0b013e318175f3a7

[10] Tredget EE Nedelec B Scott PG Hypertrophic scars, keloids, and contractures. The cellular and molecular basis for therapy. *Surg Clin North Am* 1997; 77:701–730. 9194888 10.1016/s0039-6109(05)70576-4

[11] Bishara A Nonsurgical management of hypertrophic scars: evidence-based therapies, standard practices and emerging methods. *Aesth Plast Surg* 2007; 31:468–492. 10.1007/s00266-006-0253-y 17576505

[12] Kawęcki M Bernad-Wiśniewska T Sakiel S Laser in the treatment of hypertrophic burn scars. *Intern Wound J* 2008; 5:87–97. 10.1111/j.1742-481X.2007.00309.x PMC7951724 18336382

[13] Mustoe TA Cooter RD Gold MH International clinical recommendations on scar management. *Plast Reconstr Surg* 2002; 110:560–571. 12142678 10.1097/00006534-200208000-00031

[14] Cantatore JL Kriegel DA Laser surgery: an approach to the pediatric patient. *J Am Acad Dermatol* 2004; 50:165–182. 14726870 10.1016/j.jaad.2003.08.004

[15] Bowes LE Nouri K Berman B Treatment of pigmented hypertrophic scars with the 585 nm pulsed dye laser and the 532 nm frequency-doubled Nd:YAG laser in the Q-switched and variable pulse modes: a comparative study. *Dermatol Surg* 2002; 28:714–719. 12174064 10.1046/j.1524-4725.2002.01058.x

[16] Parrett BM Donelan MB Pulsed dye laser in burn scars: current concepts and future directions. *Burns* 2010; 36:443–449. 20022430 10.1016/j.burns.2009.08.015

[17] Alster TS Improvement of erythematous and hypertrophic scars by the 585-nm flashlamp-pumped pulsed dye laser. *Ann Plast Surg* 1994; 32:186–190. 8192370 10.1097/00000637-199402000-00015

[18] Gaston P Humzah M Quaba A The pulsed tuneable dye laser as an aid in the management of postburn scarring. *Burns* 1996; 22:203–205. 8726258 10.1016/0305-4179(95)00112-3

[19] Gold MH Foster TD Adair MA Prevention of hypertrophic scars and keloids by the prophylactic use of topical silicone gel sheets following a surgical procedure in an office setting. *Dermatol Surg* 2001; 27:641–644. 11442615 10.1046/j.1524-4725.2001.00356.x

[20] Alster TS Lewis AB Rosenbach A Laser scar revision: comparison of CO_2_ laser vaporization with and without simultaneous pulsed dye laser treatment. *Dermatol Surg* 1998; 24:1299–1302. 9865192 10.1111/j.1524-4725.1998.tb00003.x

[21] Nowak KC McCormack M Koch RJ The effect of superpulsed carbon dioxide laser energy on keloid and normal dermal fibroblast secretion of growth factors: a serum-free study. *Plast Reconstr Surg* 2000; 105:2039–2048. 10839401 10.1097/00006534-200005000-00019

[22] Cheng ET Nowak KC Koch RJ Effect of blended carbon dioxide and erbium:YAG laser energy on preauricular and ear lobule keloid fibroblast secretion of growth factors: a serum-free study. *Arch Facial Plast Surg* 2001; 3:252–257. 11710860 10.1001/archfaci.3.4.252

[23] Cheng ET Pollard JD Koch RJ Effect of blended CO_2_ and erbium:YAG laser irradiation on normal and keloid fibroblasts: a serum-free study. *J Clin Laser Med Surg* 2003; 21:337–343. 14709217 10.1089/104454703322650130

[24] Clementoni MT A new treatment for severe burn and post-traumatic scars: a preliminary report. *Treat Strateg Dermatol Lasers* 2013; 2:44–48.

[25] Gould AB Gordon R. Franken PA Sands RH The LASER, light amplification by stimulated emission of radiation. *Conference on Optical Pumping*, University of Michigan, Ann Arbor, USA, 1959.

[26] Zurada JM Kriegel D Davis IC Topical treatments for hypertrophic scars. *J Am Acad Dermatol* 2006; 55:1024–1031. 17097399 10.1016/j.jaad.2006.03.022

[27] Baisch A Riedel F Hyperplastic scars and keloids. Part I: Basics and prevention. *HNO* 2006; 54:893–904. 17041777 10.1007/s00106-006-1462-z

[28] Ward RS Pressure therapy for the control of hypertrophic scar formation after burn injury: a history and review. *J Burn Care Rehabil* 1991; 12:257–262. 1885644 10.1097/00004630-199105000-00011

[29] Chang CW Ries WR Nonoperative techniques for scar management and revision. *Facial Plast Surg* 2001; 17:283–288. 11735062 10.1055/s-2001-18826

[30] Kischer CW Shetlar MR Shetlar CL Alteration of hypertrophic scars induced by mechanical pressure. *Arch Dermatol* 1975; 111:60–64. 1119824

[31] Costa AM Peyrol S Porto LC Mechanical forces induce scar remodeling study in non-pressure-treated versus pressure-treated hypertrophic scars. *Am J Pathol* 1999; 155:1671–1679. 10550323 10.1016/S0002-9440(10)65482-X PMC1866977

[32] Chang P Laubenthal KN Lewis RW II Prospective, randomized study of the efficacy of pressure garment therapy in patients with burns. *J Burn Care Rehabil* 1995; 16:473–475. 8537416 10.1097/00004630-199509000-00002

[33] Van den Kerckhove E Stappaerts K Fieuws S The assessment of erythema and thickness on burn related scars during pressure garment therapy as a preventive measure for hypertrophic scarring. *Burns* 2005; 31:696–702. 15994014 10.1016/j.burns.2005.04.014

[34] Mafong E Ashinoff R Treatment of hypertrophic scars and keloids: a review. *Aesthet Surg J* 2000; 20:114–121.

[35] Hosnuter M Payasli C Isikdemir A The effects of onion extract on hypertrophic and keloid scars. *J Wound Care* 2007; 16:251–254. 17722521 10.12968/jowc.2007.16.6.27070

[36] Jackson BA Shelton AJ Pilot study evaluating topical onion extract as treatment for postsurgical scars. *Dermatol Surg* 1999; 25:267–269. 10417579 10.1046/j.1524-4725.1999.08240.x

[37] Borok TL Bray M Sinclair I Role of ionizing irradiation for 393 keloids. *Int J Radiat Oncol Biol Phys* 1988; 15:865–870. 3182326 10.1016/0360-3016(88)90119-8

[38] Kovalic JJ Perez CA Radiation therapy following keloidectomy: a 20-year experience. *Int J Radiat Oncol Biol Phys* 1989; 17:77–80. 2745211 10.1016/0360-3016(89)90373-8

[39] Rusciani L Rossi G Bono R Use of cryotherapy in the treatment of keloids. *J Dermatol Surg Oncol* 1993; 19:529–534. 8509514 10.1111/j.1524-4725.1993.tb00386.x

[40] Zouboulis CC Blume U Buttner P Outcomes of cryosurgery in keloids and hypertrophic scars. A prospective consecutive trial of case series. *Arch Dermatol* 1993; 129:1146–1151. 8363398

[41] Har-Shai Y Amar M Sabo E Intralesional cryotherapy for enhancing the involution of hypertrophic scars and keloids. *Plast Reconstr Surg* 2003; 111:1841–1852. 12711943 10.1097/01.PRS.0000056868.42679.05

[42] Ahn ST Monafo WW Mustoe TA Topical silicone gel for the prevention and treatment of hypertrophic scar. *Arch Surg* 1991; 126:499–504. 2009067 10.1001/archsurg.1991.01410280103016

[43] O’Brien L Pandit A Silicone gel sheeting for preventing and treating hypertrophic and keloid scars. *Cochrane Database Syst Rev* 2006; 9:CD003826. 10.1002/14651858.CD003826.pub2 16437463

[44] Majan JI Evaluation of a self-adherent soft silicone dressing for the treatment of hypertrophic postoperative scars. *J Wound Care* 2006; 15:193–196. 16711171 10.12968/jowc.2006.15.5.26913

[45] de Oliveira GV Nunes TA Magna LA Silicone versus nonsilicone gel dressings: a controlled trial. *Dermatol Surg* 2001; 27:721–726. 11493295 10.1046/j.1524-4725.2001.00345.x

[46] Fulton JE Jr Silicone gel sheeting for the prevention and management of evolving hypertrophic and keloid scars. *Dermatol Surg* 1995; 21:947–951. 7582832 10.1111/j.1524-4725.1995.tb00531.x

[47] Cruz-Korchin NI Effectiveness of silicone sheets in the prevention of hypertrophic breast scars. *Ann Plast Surg* 1996; 37:345–348. 8905040 10.1097/00000637-199610000-00001

[48] Van den Kerckhove E Stappaerts K Boeckx W Silicones in the rehabilitation of burns: a review and overview. *Burns* 2001; 27:205–214. 11311512 10.1016/s0305-4179(00)00102-9

[49] Donelan MB Parrett BM Sheridan RL Pulsed dye laser therapy and Z-plasty for facial burn scars. The alternative to excision. *Ann Plast Surg* 2008; 60:480–486. 18434818 10.1097/SAP.0b013e31816fcad5

[50] Lawrence WT In search of the optimal treatment of keloids: report of a series and a review of the literature. *Ann Plast Surg* 1991; 27:164–178. 1835334 10.1097/00000637-199108000-00012

[51] Tsao SS Dover JW Arndt KA Scar management: keloid, hypertrophic, atrophic and acne scars. *Semin Cutan Med Surg* 2002; 21:46–75. 11911537 10.1016/s1085-5629(02)80719-2

[52] Eberlein A Schepler H Spilker G Erbium:YAG laser treatment of post-burn scars: potentials and limitations. *Burns* 2005; 31:15–24. 15639360 10.1016/j.burns.2004.06.004

[53] Bouzari N Davis SC Nouri K Laser treatment of keloids and hypertrophic scars. *Int J Dermatol* 2007; 46:80–88. 17214728 10.1111/j.1365-4632.2007.03104.x

[54] Kumar K Kapoor BS Rai P In-situ irradiation of keloid scars with Nd:YAG laser. *J Wound Care* 2000; 9:213–215. 11933330 10.12968/jowc.2000.9.5.25985

[55] Choi JE Oh GN Kim JY Ablative fractional laser treatment for hypertrophic scars: comparison between Er:YAG and CO_2_ fractional lasers. *J Dermatolog Treat* 2014; 25:299–303. 23621348 10.3109/09546634.2013.782090

[56] Nanni CA Alster TS Complications of carbon dioxide laser resurfacing. An evaluation of 500 patients. *Dermatol Surg* 1998; 24:315–320. 9537005 10.1111/j.1524-4725.1998.tb04161.x

[57] Campbell TM Goldman MP Adverse events of fractionated carbon dioxide laser: review of 373 treatments. *Dermatol Surg* 2010; 36:1645–1650. 20961346 10.1111/j.1524-4725.2010.01712.x

[58] Ozog DM Liu A Chaffins ML Evaluation of clinical results, histological architecture, and collagen expression following treatment of mature burn scars with a fractional carbon dioxide laser. *JAMA Dermatol* 2013; 149:50–57. 23069917 10.1001/2013.jamadermatol.668

[59] Bernstein LJ Kauvar AN Grosman MC Scar resurfacing with high-energy, short-pulsed and flashscanning carbon dioxide lasers. *Dermatol Surg* 1998; 24:101–107. 9464297 10.1111/j.1524-4725.1998.tb04060.x

[60] Qu L Liu A Zhou L Clinical and molecular effects on mature burn scars after treatment with a fractional CO2. *Lasers Surg Med* 2012; 44:517–524. 22907286 10.1002/lsm.22055

[61] Ozog D Chaffins M Moy R Histologic characteristics of mature burn scars before and after three treatments with fractional CO_2_ laser. *Laser Surg Med* 2011; 43:923.

[62] Shumaker PR Kwan JM Landers JT Functional improvements in traumatic scars and scar contractures using an ablative fractional laser protocol. *J Trauma Acute Care Surg* 2012; 73 (2 suppl 1):S116–S121. 22847080 10.1097/TA.0b013e318260634b

[63] Uebelhoer NS Ross EV Shumaker PR Ablative fractional resurfacing for the treatment of traumatic scars and contractures. *Semin Cutan Med Surg* 2012; 31:110–120. 22640431 10.1016/j.sder.2012.03.005

[64] Waibel J Beer K Fractional laser resurfacing for thermal burns. *J Drugs Dermatol* 2008; 7:59–61. 18246699

[65] Hultman CS Edkins RE Wu C Prospective, before-after cohort study to assess the efficacy of laser therapy on hypertrophic burn scars. *Ann Plast Surg* 2013; 70:521–526. 23542846 10.1097/SAP.0b013e31827eac5e

[66] Bae SH Bae YC Analysis of frequency of use of different scar assessment scales based on the scar condition and treatment method. *Arch Plast Surg* 2014; 41:111–115. 24665417 10.5999/aps.2014.41.2.111 PMC3961606

[67] Khandelwal A Yelvington M Tang X Ablative fractional photothermolysis for the treatment of hypertrophic burn scars in adult and pediatric patients: a single surgeon's experience. *J Burn Care Res* 2014; 35:455–463. 24823327 10.1097/BCR.0000000000000028

